# Supplementary material for: Are carbon emissions trading and green financial instruments synergistic? -Comprehensive quantitative research based on content analysis
Source: PLoS One. 2024 Mar 7;19(3):e0298601. doi: 10.1371/journal.pone.0298601 (PMC10919601; doi:10.1371/journal.pone.0298601)
Supplement: S1 Data — (ZIP) [file pone.0298601.s001.zip › Data/地级市Z_F工作报告.zip!/╟α║ú/╬≈─■╩╨/└┤╫╘╣1⁄2╓┌║┼í╛┬φ┐╦╩2╛▌═°í┐_2017.docx]

各位代表：

　　现在，我代表市人民政府，向大会报告政府工作，请予审议，并请市政协委员和其他列席人员提出意见。

2016年工作回顾

　　2016年，我们以习近平总书记系列重要讲话精神和“四个扎扎实实”重大要求为指引，在省委省政府和市委的坚强领导下，积极适应引领经济新常态，坚持稳中求进工作总基调，抢抓机遇、主动作为，克难攻坚、砥砺奋进，在打造绿色发展样板城市，建设幸福西宁的伟大征途上阔步前行，经济社会发展总体平稳、稳中有进、进中向好，圆满完成市第十五届人大第八次会议确定的目标任务，实现了“十三五”良好开局。盘点过去一年的工作，主要有以下五个鲜明特点：

　　——科学研判、把握大局，找准了发展新方位。市委以中国梦为根本逻辑起点，不断深化市情认识，承继“两城”建设丰富内涵，创造性提出建设幸福西宁的总目标，作出了打造绿色发展样板城市的重大部署，明确了西宁在新起点上全面建成小康社会的“目的地”“工具箱”和“路线图”，彰显西宁各族人民图发展、求突破、谋幸福的坚定决心和顽强斗志，开创了发展新境界。

　　——主动作为、行稳致远，实现了经济新增长。积极培育经济增长点、增长极和增长带，构建经济增长新格局。打好稳增长“主动仗”，经济增速逐季上扬，继续位居全国省会城市前列，总量占全省比重提高1.7个百分点，为全省发展作出了新贡献。

　　——优化布局、拓展空间，推动了城市新发展。确立“一芯双城、环状组团发展”生态山水城市发展新战略，明晰了多巴、南川、北川等重要组团和县域发展新定位，城管体制改革先行破题，“美丽夏都·清洁西宁”提升市容颜值，城市转型升级迈出新步伐。

　　——加力改革、后发赶超，培育了发展新动能。以推进供给侧结构性改革为主线，顶层设计“1+N”政策体系，“去降补”任务取得突破，在全国率先组建四级紧密型一体化医疗集团。深化“放管服”改革，构建西宁特色“一目录四清单”监管体系，释放了供给体系新活力。

　　——精准发力、补齐短板，增进了群众新福祉。紧盯率先全面建成小康社会目标，补齐农村“走教点”、基层卫生“三家服务”等公共服务短板，实施贫困家庭学生15年免费教育和大学生全程资助，同步精准帮扶城镇困难群体，统筹实施“1+8+12”精准扶贫政策体系，80个村、2.6万人脱贫摘帽，扶贫攻坚首战告捷，人民生活实现了新改善。

　　过去一年，在经济下行压力持续加大的宏观环境下，能够取得这样的成绩实属不易。我们着力抓了以下七项工作：

　　一是发展为要、多措并举，经济运行稳中有进。我们始终坚持发展第一要务，制定出台关于进一步稳定经济增长的16条措施及7个方面的配套措施，促进经济在合理区间运行。地区生产总值1248.2亿元，增长9.8%，高于全省1.8个百分点；地方一般公共预算收入75.2亿元，同口径增长9.7%；全体居民人均可支配收入突破两万元，增长9.3%。坚持供需两端同时发力，深化供给侧结构性改革，全面落实“三去一降一补”重点任务，铁合金、粗钢产量分别下降23.7%和4.7%，商品住房去化周期低于全国平均水平，减免小微企业各类税费2.5亿元；发挥投资关键作用和消费基础作用，固定资产投资1399.3亿元，增长10%，争取专项资金214.6亿元，增长13.6%。新业态、新模式加快发展，引进阿里巴巴·西宁产业带等电商运营平台，新华联、万达等知名品牌引领消费，社会消费品零售总额513.1亿元，增长11.1%，最终消费对经济增长的贡献率达到57%。

　　二是创新突破、优化结构，产业支撑夯实壮大。我们始终坚持把创新作为引领发展的第一动力，将科技创新置于优先发展的战略地位，新增科技型企业28家，高技术产业增加值增长16.1%，电子级多晶硅、光纤预制棒、锂电正极材料等技术达到国内或世界领先水平。加快调整产业结构，推动产业转型升级。工业支撑持续强化，增加值增长9.3%，开发区发挥主力军作用，规上工业增加值增长13%。新能源新材料产业加速发展，多晶硅、单晶硅和锂离子电池产量分别增长41.3%、51.9%和24.2%，夯实了光伏制造和千亿锂电基地发展基础。服务业助推经济发展，增加值增长9.3%，对经济增长贡献率46.1%，金融业增加值、旅游业总收入、电子商务交易额分别增长13%、25.2%和20%。农业保持稳定，增加值增长5.2%，高原绿色有机品牌加快培育，农村电商、乡村生态游、观光农业等成为拉动农村经济新业态。三次产业结构比为3.2：47.7：49.1，三产占比提高0.4个百分点。

　　三是科学规划、集约建设，城市综合承载力显著提升。我们始终坚持做强西宁、服务全省，着力推动城市转型升级，建设了一批打基础、利长远的民心工程，城市服务功能不断完善。充分发挥规划引领作用，编制《城市建筑风貌导则》《海绵城市设计导则》《西堡生态森林公园总体规划》等，挖掘和提炼城市特色风貌，彰显高原山水意境。畅通西宁取得突破，开工建设凤凰山快速路、西城大街等68项关键工程，成为西宁道路建设史上投资最大、开工项目最多的一年，昆仑桥拓宽改造、沈家寨及火车站互通立交等17个关键枢纽工程建成投用，外环内网交通大格局初具雏形，同步推进智慧交通、公共停车场改造、公交优先、文明出行等综合措施，交通衔接更加通畅，出行更加便捷，拥堵指数下降17.3%。不断完善市政基础设施，建成学院路等14条道路27.7公里综合管廊。建成绿道150公里。全面启动智慧西宁建设，“i西宁”APP系统上线运行。推动城市管理重心下移职能下沉，建立城市综合管理统一指挥和街道综合联动执法机制，强化街道办事处在城市综合管理中的主体地位，城市治理能力显著提升。

　　四是区域协同、城乡一体，县域城区协调并进。我们始终坚持统筹谋划县域和城区发展，构建横向差别定位、纵向分工协作的新格局。县域经济快速发展，湟中加快鲁沙尔民族文化旅游产业园和多巴新城建设，县域经济总量稳居全省第一；大通积极融入“西宁—大通沿北川河”千亿元综合产业经济带，北川省级高新技术产业园明确了主导产业方向，专业化招商破题；湟源着力建设青藏高原原产地特色产业聚集园，形成农牧业循环经济产业链条。城区经济稳步提升，城东区火车站商业核心圈加速集聚，城西区金融“洼地”效应逐渐显现，城中区南川综合片区全面启动，城北区“双创”聚集区建设成效凸显，城区辐射带动能力进一步增强。

　　五是生态优先、建管并重，人居环境持续优化。我们始终坚持“算好绿色账、走好绿色路、打好绿色牌”，深刻把握“生态似水，发展如舟”道理，着力构建“一芯两屏三廊道”城市生态屏障。强力推进生态工程建设，西堡生态森林公园完成造林4060亩，园博园项目前期和环城国家生态公园（试点）建设全面启动。“三北”“天保”、南北山三期绿化等工程造林36.1万亩。湟水河城区段建成14级清水水面。开展美化家园行动，完成307个单位庭院和居住区绿化，新增园林绿地面积1185亩。重拳治理环境污染，全面落实施工工地“五个100%”，全面完成黄标车淘汰、“煤改气”整治任务，城市空气质量优良率80.4%，提高2.8个百分点，在西北省会城市位列第一。开展湟水河沿岸养殖场搬迁，完成湟水河流域54个生活污水直排口截污纳管，湟水河出境断面水质稳定达标。全力抓好节能减排，实施工业节能改造、资源综合利用等项目82项，全面完成主要污染物减排目标，万元工业增加值能耗和用水量分别下降10%和32.8%。

　　六是以人为本、共建共享，民生福祉不断增进。我们始终坚持履职为民的坚定立场和价值取向，以小财政办大民生，民生支出占财政总支出的81%。社会事业全面进步，实施10类286个教育建设项目，完成103个农村“走教点”改造和20个乡镇幼儿园建设，城乡义务教育集团化办学改革正式启动，城市职业技术学院新校区投用。以综合医改统领医疗卫生工作，全面深化公立医院改革，完善分级诊疗制度，群众看病难、看病贵问题得到有效缓解。11所乡镇卫生院获全国“年度群众满意”殊荣。倾力保障和改善民生，城乡低保对象补助标准分别提高360元和625元，达到4836元/年和2970元/年，民生兜底保障作用效果明显。建成农村“老年之家”150个。城镇新增就业3.2万人，城镇登记失业率控制在2.48%，农村劳动力转移就业40.6万人次。建成平价蔬菜经营网点227家，居民消费价格指数累计涨幅2.1%，处于十年来最低水平。建设保障性住房4.9万套。完成1万户农村危旧房改造、75个高原美丽乡村建设。狠抓食品药品安全和安全生产工作，全年未发生食品药品和安全生产重大以上事故。深入推进平安西宁、法治西宁建设，依法妥善化解处理各类矛盾纠纷。国防动员、民族宗教、新闻广电出版、外事侨务、人防、气象、地震、供销合作、工青妇、慈善、残疾人、红十字等工作全面健康发展。

　　各位代表，在市十五届人大八次会议上，我们向全市人民承诺的十大民生实事已全部兑现，40项民生项目已全部完成！

　　七是坚持深化改革、扩大开放，发展活力竞相迸发。我们始终坚持向改革要活力、要动力，持续打造全省改革高地。简政放权创出特色，简化审批事项，优化审批流程，“一窗受理、一站服务”，建设“网上审批大厅”和“掌上审批系统”，市本级事项网上审批率达78%。着力抓好重点领域和关键环节改革，继续推进中央和省上改革试点，投融资、生态文明体制改革及医药卫生、司法、城管体制改革等稳步推进，营改增、农村宅基地制度改革和农村土地承包经营权确权登记颁证等试点有力有序，警力配置和社会治安评价机制改革成效初显，不动产统一登记稳步推进，市本级和四区公务用车制度改革全面实施。全面提升开放型经济水平，全年共引进项目306个，签约金额1144亿元，到位资金276.8亿元，比亚迪等15家全国500强企业入驻西宁。出台《西宁市“一带一路”建设行动方案》，开通国际航线2条和中欧班列，新增友好城市和地区7个，与南京市建立对口扶贫协作。成功举办首届绿色发展论坛和第十届中国国际民间艺术节，向世界展示了自信开放的西宁新形象。

　　各位代表！一年来，我们始终把建设人民满意政府作为努力方向，不断加强法治政府、廉洁政府和服务型政府建设。坚持向人大及其常委会报告工作，向人民政协通报情况，主动接受监督，共办结人大代表建议、政协委员提案270件。我们深入开展“两学一做”学习教育，持续改进作风，切实加强反腐倡廉建设，政府执行力、落实力和公信力有效提升。

　　经过全市上下的共同努力，我们的一些工作走在了全国前列：在全国第三家实行集中审批，首次明晰行政审批事项网上办理分级，率先出台政府管理事项事中事后监管清单，成为省会城市中行政审批事项最少的城市。医改工作获国务院真抓实干成效明显地方表彰，公立医院综合改革位列百家城市评估第四名。实现全国双拥模范城创建工作“七连冠”，法治政府建设位居西北第一，顺利通过“国家卫生城市”第二轮复审，成功创建全国旅游标准化示范城市，荣获“国家公共文化服务体系示范区”称号、国际友好城市交流合作奖。入选国家海绵城市建设试点、全国低碳试点城市、国家电子商务示范城市、国家医养结合试点城市、宽带中国示范城市和第二批电信普遍服务试点城市。

　　各位代表！成绩来自勠力同心，发展源于合力攻坚。西宁能有今天的发展局面，得益于中央、省委省政府和市委的正确领导，得益于市人大、市政协与各民主党派的监督支持，得益于全市人民和各级各部门同心同德、艰苦奋斗，得益于社会各界的鼎力支持、关心帮助。在此，我代表市人民政府，向全市各族干部群众，向人大代表、政协委员、各民主党派、工商联、无党派人士和各人民团体，向驻宁部队指战员、武警官兵、公安干警和驻宁企业，向所有关心、支持西宁建设发展的同志们、朋友们，致以崇高的敬意和衷心的感谢！

各位代表，冷静审视新阶段西宁发展面临的新形势、新任务、新要求，我们清醒地认识到，经济下行压力依然较大，部分企业经营困难，产业转型升级仍需加力；城市发展空间不足，绿色发展任务艰巨，城市转型升级任重道远；财政收支矛盾凸显，居民增收难度增加，经济实力支撑保障幸福西宁的能力与需要还有较大差距；政府效能还需提高，干部作风有待进一步转变等问题，需要我们在今后工作中认真加以解决。

2017年工作总体部署和主要任务

　　今年是实施“十三五”规划的重要一年，供给侧结构性改革的深化之年，打造绿色发展样板城市，建设幸福西宁的关键一年。政府工作的总体要求是：深入学习贯彻党的十八大、十九大和十八届历次中央全会以及中央经济工作会议、十八届中央纪委第七次全会精神，坚持以习近平总书记系列重要讲话特别是“3.10”、视察青海重要讲话精神和治国理政新理念新思想新战略为指导，更加自觉地增强“四个意识”特别是核心意识、看齐意识，坚定“四个自信”，始终对党绝对忠诚，坚决与以习近平同志为核心的党中央保持绝对一致，全面落实省委十二届十三次全会、省第十三次党代会、市第十四次党代会和市委十四届二次全会部署，全面践行新发展理念，全面落实“四个扎扎实实”重大要求，扎实推进“四个转变”，适应把握经济发展新常态，牢牢把握稳中求进工作总基调，统筹推进“五位一体”总体布局和协调推进“四个全面”战略布局，按照市委“12315”总目标，着力深化供给侧结构性改革，着力推动绿色发展，着力深化创新驱动，着力补齐发展短板，着力保障和改善民生，推动绿色发展样板城市和幸福西宁建设取得突破性进展，以优异成绩迎接党的十九大和省第十三次党代会的胜利召开。

　　主要预期目标是：地区生产总值增长9.5%左右，固定资产投资增长10%，规模以上工业增加值增长9%，社会消费品零售总额增长10%，地方一般公共预算收入同口径增长8%，全体居民人均可支配收入增长9%以上，城市空气质量优良率75%以上，单位GDP能耗、CPI、主要污染物排放完成省上下达的控制指标。上述预期目标，既坚持稳中求进工作总基调，力促经济“行稳致远”，又发扬“跳起来摘桃子”精神，为“十三五”后几年的发展蓄势减压，突出稳与进的辩证统一，彰显了省会城市责任担当。

　　实现上述奋斗目标，我们必须按照省委省政府“建设美丽西宁，服务全省人民”和“发挥西宁引领全省发展‘火车头’作用”要求，紧紧抓住供给侧结构性改革一条主线，突出稳增长、优生态、惠民生三大领域，把握产业和城市转型升级两个着力点，全力以赴打好五大攻坚战。

　　——全力以赴打好脱贫攻坚战。紧盯率先实现全面建成小康社会目标不动摇，走在前、赶在先，确保年内100个村、2.5万人口脱贫摘帽，为2018年全面脱贫打好基础。

　　——全力以赴打好结构调整攻坚战。紧盯供给侧结构性改革新要求不放松，调结构、促转型，强化创新驱动，振兴实体经济，着力提升城市产业竞争力。

　　——全力以赴打好畅通西宁攻坚战。紧盯提升城市治理能力不减力，强基础、补短板，加快实施“畅通西宁”三年攻坚行动计划的惠民便民工程，构建“外环内网”交通大格局。

　　——全力以赴打好创建全国文明城市攻坚战。紧盯创建全国文明城市目标不停步，找差距、攻难点，坚持一城抓总、多城联创，塑造城市新形象，铸造全国文明城市“金名片”。

　　——全力以赴打好环境保护攻坚战。紧盯“生态优先”与“发展率先”两个关键不懈怠，强督查、促整改，将生态建设、环保监管作为示范工程和关键环节，把绿色发展的根扎深扎牢。

　　围绕五大攻坚战，力争在八个方面再上新台阶：

　　（一）持续扩大有效投资，夯实筑牢发展底盘，力争项目建设再上新台阶。继续把扩大有效投资作为稳增长的主支撑，积极争取、精准对接一批支撑作用大、带动能力强、市场前景好的大项目、好项目，调动民间投资积极性，扩大招商引资规模，确保固定资产投资增长10%。

　　优化结构扩大规模。在扩大投资规模的同时，不断优化投资结构，提高投资质量和效益，有效拉动经济增长。发挥工业投资的支撑作用，围绕光伏制造和千亿锂电两个基地、铝镁合金、装备制造、生物医药等谋划引进项目，加快项目落地，全力稳定工业投资。突出基础设施投资“稳定器”作用，把投资重点放在重大片区规划落地、畅通西宁、城乡基础设施建设等重点领域，开工一批重大项目，不断增强城市承载能力。保持房地产投资稳定增长，加快南川、北川重点片区土地开发利用，引进一批品牌企业，促进房地产业提升品质、打造品牌。不断扩大民生领域投资，加大教育、医疗、养老、扶贫等重点领域和薄弱环节的投资力度，促进公共服务均等化。

　　强化重大项目协调督导。项目督导不松懈，紧盯今年180项重点项目，严格执行周检查、旬协调、月督办的项目调度制度，强力推进项目落地见效，力争续建项目3月份复工率达到90%以上、新建项目上半年开工率达到70%以上。项目储备不懈怠，围绕“十三五”规划、“一带一路”倡议、国家适度扩大总需求的重点投向，谋划一批拉动力强、利长远、增后劲的重大项目，与国家专项建设基金申报争取联动互补，建立连续不断、滚动实施的项目储备库。项目融资不松劲，发挥财政资金的引导和撬动作用，加大专项资金、地方政府债券、专项建设基金等争取力度，用活用好中央和省市各类财政资金，提高政府投资的精准性和有效性。加强与金融机构合作，开展融资租赁、资产证券化、多层次资本市场直接融资等方式扩大融资规模。动态调整PPP滚动项目库，加强与中国PPP基金的衔接，加快项目对接，吸引更多社会投资。

　　提高招商引资实效，扩大民间投资。坚持把招商引资作为重中之重，继续坚持市级领导带队招商、全员招商，用好青洽会、城洽会、南京对口帮扶等平台机制，通过产业链招商、猎头式招商、商会集团式招商、研发机构推荐招商等新模式，对准九大重点产业链，做好铝合金、光伏光热、锂电、生物医药、文化旅游、基础设施与房地产等方面的全面推介招商，着力抓好王府井集团、正威、修正药业等知名企业招商对接，年内招商引资到位资金880亿元。全面落实支持民间投资发展的政策措施，制定我市支持民间投资发展实施方案，从项目准入、融资平台、政策支持等方面加大扶持和保障力度，规范中介服务，提振民间投资信心。同时，加大对园区基础设施建设的协调服务力度，全力抓好要素保障。

　　（二）推进农业供给侧结构性改革，补齐全面小康短板，力争“三农”工作再上新台阶。围绕市场需求，以增加农民收入、保障有效供给为目标，提高农业供给质量，激活市场、要素、主体，优化农业产业、生产、经营体系，提高土地产出率、资源利用率、劳动生产率。农业增加值增长5%，农村常住居民人均可支配收入增长9%以上。

　　打好脱贫攻坚战。重点实施好4万贫困人口产业帮扶、1200户易地搬迁、大通县扶贫产业园等项目。把高原美丽乡村建设、乡村旅游开发与脱贫攻坚结合起来，整合资源、集中投入，推进农业、林业与旅游、教育、文化、康养等产业深度融合，建设一批农业文化旅游“三位一体”、生产生活生态同步改善、三次产业深度融合的乡村旅游扶贫特色村，建设乡村旅游示范带。抓紧抓实与南京的对口帮扶工作，在产业融合协作、人才交流培训等方面积极对接、争取支持，合力打赢脱贫攻坚战。

　　推进农业供给侧结构性改革。实施耕地质量保护与提升行动，确保实有耕地数量稳定、质量提升。探索建立农业农村发展用地保障机制，完善农村土地“三权”分置办法，完成耕地确权登记颁证任务，盘活农村存量建设用地，统筹推进湟源县“国字号”三项试点任务，为各项农村改革打牢底盘。有序推进农村集体经营性资产股份合作制改革，全面开展农村集体资产清产核资，完成湟中县农村集体资产股份权能改革试点任务。深化涉农投融资体制机制改革，制定农村金融配套改革方案，依托市级信保集团，健全县乡综合信用担保体系，引导金融机构下沉金融网点和服务，推进农村产权抵押贷款试点。发展多种形式的农业适度规模经营，大力培育新型农业经营主体和服务主体，支持有条件的乡村建设集循环农业、创意农业、农事体验于一体的田园综合体，专项资金以股权方式投入农业经营主体。

　　大力发展绿色有机品牌农业。以规模化种养基地为基础，依托农业产业化龙头企业带动，聚集现代生产要素，吸引龙头企业和科研机构建设运营产业园，建设“生产+加工+科技”的现代农业产业园，巩固提升大通县国家级现代农业示范区，推进湟源、湟中县国家级现代农业示范区创建，建设一批市级现代农业产业示范园。大力发展中藏药材、饲草等特色农业产业，整合加工资源，构建种加销一体化发展格局，推动农业全环节升级、全链条增值。以增加绿色优质农产品供给为主攻方向，发展有机栽培、水肥一体化等绿色种植技术，继续实施养殖业出川上山，建设万亩生态牧场6家，严格化肥、农药、饲料添加剂等使用管理，落实绿色产品标准、认证体系，打造高原绿色有机品牌。

　　多渠道增加农民收入。培训新型职业农民3000人，培育劳务输出品牌，组织好园区和建筑业用工对接，农村劳动力转移就业35万人次以上，稳定工资性收入。鼓励农民合作联合、龙头企业带动，大力发展乡村旅游、庭院经济、农机出租等服务业，提高经营性收入。激发农村资源资产要素活力，流转农村土地44万亩，通过土地股份合作、托管代耕等多种经营形式，增加财产性收入。落实好农机具购置补贴、新型农村养老保险、低保等强农惠农富农政策，保障政策性收入。发展农村普惠金融，开展农民合作社内部信用合作试点，鼓励发展农业互助保险。通过“金穗服务惠万家”“双基联动”等信贷支持，为有需求的农户提供贴息贷款，降低融资成本。

　　（三）发挥工业对稳增长的主力军作用，大力振兴实体经济，力争工业经济再上新台阶。坚持绿色循环低碳方向，改造提升传统产业与培育壮大新兴产业并举，扩总量、提质量、优结构，促进工业经济转型升级。

全面落实“三去一降一补”任务。以严格的环保能耗、质量安全等标准，倒逼企业兼并重组。继续推动煤炭、水泥等行业化解过剩产能，关闭退出大通煤矿120万吨落后产能，化解100万吨低标号水泥等过剩产能。加快本地企业产销对接，提高地产工业品采购比例，推进铝水直供，提高电解铝就地转化率。落实好国家金融政策，引导企业优化资本结构，加大股权融资力度，加强企业自身债务杠杆约束等，降低企业杠杆率。加强金融机构与重点企业、重点项目的对接合作，建立“过桥”资金池，为重点领域企业提供信贷周转、融资贴息、担保补贴等，帮助企业降低信贷成本。

　　打好结构调整攻坚战。深入实施中国制造2025青海行动方案，推动优势和战略性新兴产业快速崛起，形成若干新的“增长点”，推动制造业向中高端迈进。打造特色传统产业竞争新优势，实施“百项改造提升工程”，抓好电解铝生产线改造、电池组件扩建改造等116项技改项目，推动传统产业向高附加值环节攀升。稳定电解铝生产，抓紧高端铝材等产业链延伸项目建设，加快铝镁合金高新材料加工产业聚集；利用“互联网+”技术创新商业模式，提升藏毯绒纺产业发展水平。培育壮大新兴产业，以新能源示范省建设为牵引，落实好“百项创新攻坚工程”、光伏制造业基地建设实施意见和千亿元锂电产业基地建设实施方案，迅速扩大新兴产业规模。推动上游生产制造与下游电站建设联动，构建垂直一体化光伏产业发展新模式，全力打造全国重要的光伏制造中心。加快构建完整锂电产业链，大力发展锂电配套产业，推进比亚迪动力锂电池、华泰锂电池及新能源汽车基地、北捷锂电池隔膜等关键项目建设，打造全国重要的千亿锂电产业基地。加快普兰特中药颗粒、九康中药饮片等项目建设步伐，提升超临界萃取沙棘油能力，力争申报地黄提取梓醇产品为全省首个国家一类新药，打造高原特色地方资源精深加工和中藏药产业基地。

　　全力以赴稳定工业增长。密切监控生产经营趋势动态，加强运行分析和监测预警，切实提高地区工业经济运行的调控能力和水平。做好百和再生铝、开源金属等企业启动负荷工作。扩大现有企业产能，重点抓好国电投太阳能电池组件、黄河水电新能源多晶硅等项目建设，力争早日形成新的增长点。发挥好产业基金引导作用，加大对重点行业企业支持力度。

　　促进工业经济转型升级。实施“百户领军企业打造工程”，培育入规工业企业15户以上，新增销售5亿元以上企业5户以上、10亿元以上企业2户以上。狠抓千家中小微企业培育，发展一批成长型、创新型小微企业，新增中小微企业6000户以上。引导企业形成自己独有的比较优势，发扬“工匠精神”，加强品牌建设，提高质量标准，增强产品竞争力。

　　（四）坚持城区县域联动，促进区域协调发展，力争城乡一体再上新台阶。围绕建设西宁周边1小时经济生活圈，促进城乡对接，强化优势互补，着力加强市县基础设施互联互通、公共服务设施共建共享，推动中心城区和县域经济发展“双轮驱动”，推进城乡协调联动、双向发展。

　　发挥城区核心引领作用，促进服务业转型升级。巩固国家服务业综合试点城市成果，发挥城区服务业集聚示范效应，辐射带动县域增强综合服务能力。巩固旅游业快速发展势头，充分发挥中国西部自驾车旅游联盟聚集作用，继续提升服务水平，进一步强化西部旅游集散功能，游客接待人数增长13%，旅游总收入增长20%。积极规划建设海洋馆、冰球馆，推进新华联国际旅游城、可可西里文化旅游产业园、乡村休闲游等带动性强、辐射广、影响远的项目。启动通用机场建设前期工作，促进通用航空与旅游、体育以及互联网、创意经济融合发展，引领新兴大众消费。积极发挥新消费引领作用，加快培育九大类生活性服务业，推动商贸服务业高端化，重点引进红星美凯龙、奥特莱斯等新型业态及品牌商业，依托万达广场、水井巷商务区等商贸设施，打造特色商业集聚区。建设多功能乡镇商贸中心，推动品牌连锁超市入驻，畅通农村商贸流通网络。申报现代物流创新发展试点城市，开展肉类及农产品冷链物流仓储、配送试点，加快朝阳物流园信息化改造升级，把丝绸之路（国际）物流城建成向西开放的大型物流基地。统筹引导市区物流、建材、屠宰、批发和汽配等市场向三县适合区域转移。加快城西现代金融聚集区建设，组建市金融控股集团，推动“稳当宝”等互联网金融平台规范运营。支持养老、养生、家政等生活性服务业态繁荣发展。支持电子商务新业态发展，完善电子商务政策法规环境和服务支撑体系，建设国家电子商务示范企业2家，积极创建国家电子商务示范城市。与阿里巴巴集团合作，建成100个示范性新型农村电商淘宝店，建设湟中、大通国家电子商务进农村综合示范县，培育西宁知名电商品牌。

　　补齐补强县域经济发展短板。着力挖掘县域经济发展新空间、新动力、新优势，用改革的办法、市场的手段激发县域经济内生动力，促进人口和产业聚集。依托园区发展县域主导产业，把园区作为县域经济发展的主战场、项目建设的主平台，更好更快地促进项目落地和产业聚集。大通县依托北川省级高新技术产业开发区加快打造铝镁合金等高新材料产业集群，延伸铝精深加工产业链。湟中县以多巴新城建设为重点，加快与主城一体步伐，推动行政区划调整，发挥鲁沙尔民族文化旅游产业园、甘河工业园带动优势，加快由经济大县向经济强县转变。湟源县发挥背靠大牧区、面向大市场的地缘优势，依托全省农畜产品资源，大力发展高原绿色食品加工业，加快建设青藏高原原产地特色产业聚集园。将民营经济作为县域经济发展的突破口，以市场化方式建设县域产业园和中小微企业创业基地，着力孵化和培育市场主体，培育县域民营中小企业360家，让民营经济带动县域经济发展。以特色小镇为抓手促进县域经济创特色，挖掘产业特色、人文底蕴和生态禀赋，以乡村旅游示范带建设为抓手，培育休闲旅游、商贸物流、特色制造、民俗文化等特色小镇。大通县依托鹞子沟国家森林公园，打造东峡镇休闲旅游特色镇，借鉴南京江宁模式打造朔北窎沟片区乡村旅游产业园；湟中县加快建设陈家滩特色文化产业园，打造鲁沙尔旅游风情小镇，围绕云谷川和西纳川打造卡阳乡趣生态旅游区；湟源县以丹噶尔古城保护开发为核心，打造历史文化名城，建设高原旅游名镇。

　　推动城乡基础设施互联互通。统筹城乡基础设施布局与建设，提高城区与县域同城化发展辐射带动力。强化交通功能引领，开展市域国省干线公路、城市道路、县乡公路交通衔接布局研究与规划，优化“三环七射”高速公路网布局，协调推进227国道南北段、昆仑大道西延段、G6高速北移、大湟平公路等建设，按领先全省的标准升级改造县乡道路、联村道路，构建城区与三县更加便捷的路网体系，推进城乡交通一体化。实施园博园及西堡生态森林公园水利灌溉项目，完成农村饮水安全巩固提升、水土保持及小流域治理年度建设任务。提升城市配电网供电可靠性，改造升级35个中心村电网。加快通信基础设施及物联网建设，实施信息惠民工程。有效整合高原美丽乡村建设项目，完善农村医疗、文化、体育等基础设施。引导农民就近向城镇集中，加快形成以城带乡、城乡互动的发展格局。

　　（五）提升规划建设管理水平，加快重点片区建设，力争城市面貌再上新台阶。加快城市转型升级步伐，主动对接国家兰西城市群发展规划，落实东部城市群建设战略部署，推动城市发展向组团化疏解、内涵式提升转变。

　　精心规划城市。启动市域空间规划“多规合一”试点工作，构建全域统一衔接、人与自然和谐相处的绿色空间规划体系。启动新一轮城市总体规划修编前期研究，推进行政区划调整。申报国家级产城融合示范区及“生态修复城市修补”试点城市，启动编制各园区产城融合专项规划和城市“双修”总体规划、专项规划。加快西宁卫城传统特色街区建设，努力打造具有现代人文气息，蕴含丰富文化底蕴的高原山水生态城市。严格执行城市建筑风貌导则，提高规划建设理念标准，塑造一批河湟精品建筑，彰显独特地域风貌。建设海绵试点城市，将低影响开发理念贯穿于各层次规划和各类开发建设活动，确保今年完成70%的建设任务。申报省级城市设计试点城市，分级分层推进总体、分区、重点区域城市设计，实现城市轴线、天际线、文脉、风格和色彩的协调统一。全面梳理城市管线，有序推进城市地下管线升级改造，启动城市架空线整治入地工程。

　　精准落实重点片区规划。围绕多个经济增长点、三个经济增长极和三条千亿元经济增长带的培育，加快推动“十三五”规划确定的重点片区建设，促进重大项目落地。多巴新城突出生态山水城市风貌特色和城市副中心定位，完成总体规划编制报批，加快建设地下综合管廊和地上骨干交通体系，启动实施周边沟道生态治理和“南北两山”绿化工程，打造国家新型城镇化试点的示范区。南川片区加快完成南川文化旅游商贸会展区城市设计+风貌导则和控制性详细规划，启动市民中心、南川高级中学、青大附属医院等项目，抓好文化旅游会展综合体等公共服务设施项目前期，以文化商贸旅游会展业带动城市均衡发展。北川片区落实好铝镁合金高新材料产业园规划，加快组建铝镁合金高新材料研发中心，加大路网等基础设施建设，着力构建以铝镁合金高新材料集群为主导的循环经济产业体系。西堡生态森林公园编制完成道路、水利、电力等专项规划，启动绿化道路建设，抓好灌溉系统配套设施建设，完成2.8万亩绿化造林任务。

　　精致建设城市。打好畅通西宁攻坚战，新开昆仑路地下通道、长江路拓宽改造等22个项目，年内实现90%以上外环内网项目开工，峡口连接线、湟水路高架桥等18项工程竣工。新增（优化）18条公交线路，建成公交9路线公交专用道；开工城南等地区大型地下停车场，继续改扩建一批公共临时停车场，进一步规范停车收费；在行人过街需求集中部分路段建设人行天桥；基本完成智慧交通管理系统，提高信号控制、路况监控等智能化水平，提升道路交通秩序精细化治理能力；新建绿道100公里，构建连接中心城区和三县的生态绿道网络。增强基础设施支撑能力，启动城市南部水源工程前期，加快西纳川水库、“引大济湟”西干渠建设，缓解园区工业和周边城镇用水、灌溉及生态缺水问题。开展综合管廊二期建设。加快城市轨道1号线前期工作步伐。积极配合省上实施好西宁机场三期、西成铁路、城市电网等重大项目建设。建成智慧城市综合运行管理指挥中心，完成统一的政务云平台、大数据基础平台、视频资源共享平台，加快智慧旅游、智慧卫生等项目建设，推动“互联网+服务”。打造完善“i西宁”城市门户民生服务客户端等“七个一”民生工程，让群众少跑腿，让信息多跑路。

　　精细治理城市。着力提高城市精细化管理和公共服务水平，推动城市管理向城市治理转变。深化城管体制改革，健全完善街道城管综合执法中心，做实街道城市综合管理的主体责任，加强考核评比，严格监管督导，建立统一的市政公用设施抢修维护体系，提升管养水平。持续打造“美丽夏都·清洁西宁”品牌，开展小街小巷机械化作业试点，落实“门前三包”和“严管重罚”措施。开展“无人机”巡查，对重点区域实行实时动态监控。解决现有垃圾填埋场和粪便无害化处理厂遗留问题，规范垃圾填埋场管理运营，加快推进垃圾焚烧和生活垃圾分拣及资源化利用项目建设。开展乡镇村垃圾收集处理和建制镇污水收集处理体系建设，实现城镇生活污水和乡镇村垃圾全收集、全处理。以农村环境综合整治为抓手，围绕县城周边、国省道公路沿线和旅游景区，建设一批村容整洁、生态美好、乡风文明的示范村和连片示范区。打好创建全国文明城市攻坚战，利用微信、媒体、政务热线等平台监督曝光不文明行为，建立不文明行为有奖举报和处罚制度，严管重罚乱吐乱扔、乱停乱放，营造城市环境共建共享的浓厚氛围。开展城乡接合部和城市出入口专项整治，对乱倒垃圾、乱堆物资、占道修洗车等进行集中整治。加强对流浪乞讨人员的救助，规范出租车运营，强化文明劝导员、志愿者作用，不断提高市民文明素质，树立城市文明形象，全力冲刺，力争成功创建全国文明城市。

　　（六）加强生态环境建设保护，打造生态文明高地，力争绿色发展再上新台阶。坚定不移加强生态保护与建设，推进生产循环化、生活低碳化、治理现代化，将幸福西宁书写在青山绿水之间，让生态秀美成为市民的永续福利。

　　加快生态建设步伐。坚守好生态涵养绿色本底，着力开展河岸线和山体生态修复，打造南北山绿化示范工程，完成重点工程造林33.2万亩，全面启动西堡生态森林公园、园博园、德促贷款绿化和生态保护项目，推进环城国家生态公园（试点）建设，森林覆盖率达到33%。大力实施城市绿化美化，持续开展美化家园绿化行动，完成人民公园、长青园等景观改造提升，新建苦水沟、湟岸巷等10处街头绿地，提升93公里绿道绿化景观，持续推进单位庭院和居住区绿化达标行动，全年新增园林绿地600亩。全面推进水生态文明城市建设，扎实做好水生态廊道连通综合功能提升、南川河、黑泉水库水生态文明建设等项目。全面落实河长制，大力推进西川河生态综合治理工程，打造“三河六岸”绿色景观生态廊道。

　　打好环境保护攻坚战。以中央和全省环保督察为契机，坚持问题导向，摸排隐患，全面整改问题，强化问责，提升实效。铁腕治理大气污染，加密空气质量监测点，构建网格化空气质量监控体系。继续推行“五个100%”的扬尘管控措施，强化工业污染治理，完成150蒸吨“煤改气”、4300辆黄标车淘汰任务，确保空气质量只升不降。强力推进水污染治理，新建东川工业园工业废水处理厂，做好多巴生活污水处理厂前期，加快六污新建、四污扩能改造、五污中水回用和甘河西区工业污水处理项目。加快湟水流域企业排水和生活污水截污纳管，确保小峡口出境断面水质持续稳定达标。启动农村饮用水水源地整治工程。坚决消除固废污染，强化危险废物产生单位、经营单位监管，实行医废应收尽收无害化焚烧处置，实施铬渣污染场地治理修复项目。严厉打击环保违法行为，全面落实环境保护主体责任，加强环境基础能力建设，继续深化环保执法与司法联动，重拳打击环境违法行为。

　　推动绿色低碳循环发展。积极做好国家低碳城市试点各项工作，构建具有西宁特色的低碳发展模式。开展东川、甘河、南川工业园区国家循环化改造示范试点和低碳示范试点，构建绿色低碳循环发展产业体系。加强工业、建筑等重点领域节能管理，提高园区排放标准，关闭一批高排放企业，推行钢铁、电解铝、光伏等行业能效对标达标活动，实施燃煤锅炉节能环保综合提升、既有建筑节能改造、公共建筑效能提升、绿色照明、余热暖民等节能重点工程。大力发展绿色金融，以金融手段激励企业主动减排。落实好《西宁市新能源汽车推广应用实施方案》，加快充电桩等设施建设，积极推广新能源汽车。把生态文明建设纳入制度化、法治化轨道，构建促进绿色发展的体制机制。建立完善全面体现绿色发展样板城市建设要求的经济社会发展评价体系、考核办法和奖惩机制。

　　（七）聚焦聚力热点难点，倾力保障和改善民生，力争社会事业再上新台阶。继续秉承和坚持以人民为中心的发展思想，对标全面建成小康社会要求，坚持财政资金优先保障民生投入，加快补齐民生短板，让广大群众拥有更多的获得感和幸福感。

　　繁荣社会事业。进一步优化调整教育布局，启动消除城镇大班额专项计划，深入实施义务教育“全面改薄”和标准化学校建设，积极推进集团化办学，逐步扩大15年免费教育政策实施范围，继续对贫困家庭大学生在校期间学费和生活费实施全程资助。全面实施《健康西宁2016—2020行动计划》，推动健康事业与健康产业有机衔接，推进医养结合示范市建设。开展精准扶贫县乡村卫生机构设备提升工程，为县级医院配发医疗设备，为51所乡镇卫生院、503所行政村、扶贫村卫生室更新医疗设备。设立计划生育困难家庭救助金。抓好食品安全示范城市创建工作，打造10家全面落实食品安全主体责任的示范流通企业，加快建立食品安全“快检速查”网络监管体系，健全从生产到消费全过程监管链条，严守从农田到餐桌每一道防线。加快文旅融合，大力发展原生态文化产业，在产业基金引导、文旅集团运营、精品剧目打造等方面迈出实质性步伐。加快推进四级公共文化基础设施建设，新建和改扩建部分县区图书馆、文化馆（群艺馆）和博物馆。办好环湖赛西宁赛段、国际徒步节、市运会等活动，不断扩大知名度和影响力。

　　促进社会和谐。扎实推进平安西宁、法治西宁建设，完善社会治安防控“六张网”，构建以信息化为支撑的社会治安立体防控体系。全面深化公安改革，推动社会治安治理方式从末端处理向前端治理转移，全力打造良好社会治安生态。严格落实安全生产“党政同责、一岗双责”和“事前问责”要求，狠抓隐患排查治理和专项整治，坚决防范和遏制重特大安全生产事故发生。深入开展双拥共建，促进军民融合发展。深化法治县（区）、民主法治示范村（社区）等创建活动，不断提高基层治理法治化水平。进一步巩固团结互助、平等友爱、和睦融洽的民族关系。关心支持工会、共青团、工商联、红十字会、妇女、老龄、儿童、残疾人工作，积极推进仲裁、防震减灾、人防等事业发展。

　　夯实民生保障。完善市、区（县）孵化基地及产业园区间促进创新创业发展孵化服务联动机制，城镇新增就业人数3万人，城镇登记失业率控制在2.5%以内。稳步提高城乡居民医保筹资标准和保障水平，继续扩大社会保险覆盖面。完善城乡社会救助体系，统筹做好城乡低保、医疗救助、临时救助、五保户供养、优抚安置和残疾人工作。发展适度普惠型社会福利事业，打造“1+7+N”三级养老服务体系，加快建设省、市养老示范基地和儿童福利院。继续实施政府购买养老服务，在每个县区建设一个养老示范基地，在农村建设50个农村老年之家，在城市规范运营100个社区日间照料中心。强化保障性住房建设分配和运营管理，建设保障性住房18690套，完成6500户农村危旧房改造。高度关注全年物价走势，发挥国有农产品控股流通公司引导作用，新建平价菜店60个，增强政府物价调控能力。

　　各位代表！民生连着民心，我们要以百姓之心为心，倾情关注困难群众，多做雪中送炭的民生实事，一件一件抓落实，千方百计把群众日常生活的琐事、小事、烦心事办好做实。今年市委市政府将集中力量继续实施一批解民忧、疏民困的民生实事项目，在新的一年里为人民群众创造更多福祉，努力让全市人民生活得更加美好更加幸福。

（八）全面深化改革创新，竞相激发活力动力，力争开放合作再上新台阶。毫不放松抓好改革开放，以改革添活力、以开放增动力，加快实施创新驱动发展战略，进一步补齐发展短板、激发发展活力、厚植发展优势。

以更大力度实施创新驱动战略。强化“弱鸟先飞”意识，切实把创新驱动作为优先战略，深化科技体制改革，建设国家创新型试点城市，以科技创新引领绿色发展样板城市建设。发挥创新聚集作用，继续推进新能源新材料、高端装备制造、特色生物资源精深加工等重点领域技术创新。扎实推进科技型企业和高新技术企业“两个倍增”工程及科技“小巨人”培育计划，培育科技型企业20家、高新技术企业18家。完善机制，加大人才培养引进力度，汇聚科技创新、企业管理等方面的拔尖人才，发挥人才在创新驱动中的引擎作用。与中色科技股份有限公司合作建立技术创新战略联盟，发挥浦东·西宁科技合作交流中心等“科技创新飞地”作用。加快建设西宁科技大市场和科技服务业集聚区。

　　以更大决心深化重点领域改革。深化“放管服”改革，继续优化行政审批流程，建成“一网多端”行政审批网上办事大厅，完善事中事后监管，努力降低制度性交易成本。深化国资国企改革，落实市属国资国企改革方案，开展经营业绩考核，完善企业法人治理结构，提升国企管理水平。稳妥推进财税体制改革，全面完成深化预算管理制度改革各项工作。落实好税收制度改革政策，推进地方税体系建设。深化医药卫生体制改革，继续拓展四级紧密型一体化医疗联合体建设，健全分级诊疗制度，形成以付费总额控制为主的复合型支付模式。积极推动社保体系改革，全面落实机关事业单位养老保险制度改革相关政策。

以更宽视野扩大对外开放。充分发挥西宁连接“一带一路”和长江经济带特别是面向中亚开放前沿的枢纽地位，突出西宁的门户地位、集散功能，以西部自驾游联盟为龙头，推动旅游业由低端观光向高端体验升级，努力打造丝绸之路经济带特色旅游名城、中国西部区域旅游集散中心和青藏高原国际旅游服务基地。推动内外贸投资融合发展，加快建立“扩出口”与“促消费”相结合、外贸企业扩内需等政策机制。推行“保税仓储+保税展销”模式，继续建设进口商品馆和保税购物中心，启动西宁综合保税区建设。加快建设中国（西宁）国际绿色产业园，发挥好土库曼斯坦采购及商贸中心等平台作用。着力建设南川文化旅游商贸会展、北川特色口岸综合服务等具有国际影响力的开放支撑平台。

　　各位代表，让人民群众生活得更幸福，把西宁建设得更美好，是我们的神圣职责和光荣使命。我们要始终把为人民谋发展增福祉作为最大责任，始终把群众冷暖忧乐放在心头，始终把建设幸福西宁的使命扛在肩上，认真落实中央八项规定精神和省市各项措施，坚持不懈纠正“四风”问题，持续开展懒政怠政为官不为专项治理，大力整治行政不作为、慢作为、乱作为现象，严肃惩治不正之风和腐败行为，切实提高政府施政能力和服务水平。

各位代表，新的目标蓝图，激励我们筑梦前行；人民的信任重托，鞭策我们勇往直前。让我们更加紧密地团结在以习近平同志为核心的党中央周围，始终对党绝对忠诚，坚决与以习近平同志为核心的党中央保持绝对一致，在省委、省政府和市委的坚强领导下，紧紧依靠全市各族人民，为打造绿色发展样板城市、谱写中华民族伟大复兴中国梦幸福西宁篇章而不懈奋斗，以优异成绩迎接党的十九大和省第十三次党代会的胜利召开！
